# Nonlinear Vibration of Double-Walled Carbon Nanotubes Subjected to Mechanical Impact and Embedded on Winkler–Pasternak Foundation

**DOI:** 10.3390/ma15238599

**Published:** 2022-12-02

**Authors:** Nicolae Herisanu, Bogdan Marinca, Vasile Marinca

**Affiliations:** 1 Department of Mechanics and Strength of Materials, University Politehnica Timisoara, 300222 Timisoara, Romania; 2 Department of Applied Electronics, University Politehnica Timisoara, 300223 Timisoara, Romania

**Keywords:** double-walled carbon nanotubes, mechanical impact, optimal auxiliary functions method, approximate solutions, stability, Winkler–Pasternak foundation

## Abstract

This study was devoted to an investigation on the dynamics of double-walled carbon nanotubes (DWCNTs) under the influence of Winkler–Pasternak foundation near the primary resonance. Two Euler–Bernoulli beams embedded on nonlinear foundation, interacting through van der Waals forces, subjected to mechanical impact are considered. By means of Hamilton’s principle, Eringen’s nonlocal elastic theory, and taking into account the moving nanoparticles, the Galerkin–Bubnov method is applied and accordingly, governing partial differential equations are reduced to two differential equations with variable coefficients. The nonlinear damped and forced vibration is studied using the optimal auxiliary functions method (OAFM). An explicit and very accurate analytical solution is obtained by means of OAFM without considering simplifying hypotheses. An accurate analysis is for the first time reported considering the cumulated effects of nonlinearities simultaneously induced by the Winkler–Pasternak foundation, the curvature of beams and van der Waals force, and also the effect of discontinuities marked by the presence of the Dirac function. Finally, a stability analysis of the considered model is developed by means of the homotopy perturbation method (HPM) using the condition of existence of the two frequencies. It was shown that an increasing of some constitutive parameters substantially reduces the area of stability, all these being of much help in guiding the design of advanced nanoelectromechanical devices, in which nanotubes act as basic elements.

## 1. Introduction

Starting with the discovery of carbon nanotubes (CNTs) by Iijima [1] in 1991, much research has been conducted on this new attractive topic in the field of nanotechnology. CNTs have a lot of applications in atomic-force microscopy, gas storage, fluid transport, nanosprings, nanofillers, nanomotors, agricultural or chemical industry, nanoelectromechanical devices, fuel cells, thermal and electrical conductivities, and so on. Slender or functionally graded Euler–Bernoulli, Timoshenko, Reddy, or Rayleigh beams, linear or nonlinear with Pasternak or Winkler foundation with or without van der Waals interactions, and free or forced vibrations were investigated in the last few years. These studies were basically conducted in two different principal ways delimited by considering or not the nonlinearity and associated nonlinear effects. In the first approach, nonlinearity and nonlinear terms were omitted, and in this first category of studies, a closed-form solution for the vibration of a linear carbon chain in CNTs was derived by Ding et al. [2] by means of continuum modeling of the van der Waals interactions. Analytical results showed that the vibration frequency of the carbon chain in a (5,5) CNT could be around two orders of magnitude higher than that of an independent carbon chain without initial tensile force. Rezapour and Araghi [3] considered the effects of inertia and Coriolis forces due to nanoparticle movement and viscoelastic behavior of single-walled carbon nanotubes (SWCNTs) is established by the Kelvin–Voigt viscoelastic model. The dynamic amplitude was found in this study almost 30% higher than the corresponding value obtained from the moving load model. The incremental harmonic balance method was implemented by Pirmoradian et al. [4] to investigate the parametric resonance of double-walled carbon nanotubes (DWCNTs) loaded by successive nanoparticles through the drug-delivery process. Comparing the static tensile axial force with the case of compressive force, they concluded that the DWCNT is more stable in the first case. Moreover, the instability of transverse vibration of an embedded DWCNT for the delivery of successive nanoparticles was investigated by Pirmoradian et al. [5] considering the van der Waals force and inertial effects of moving nanoparticles, and stability was determined by the incremental harmonic balance method.

A more realistic approach closer to the reality is obtained by considering also the nonlinearity in modeling these kinds of problems. Such nonlinear models were investigated by different researchers using various analytical techniques applicable in analyzing nonlinear differential equations. Soltani and Farshidianfar [6] applied the energy balance method in the study of vibrations of SWCNTs embedded on Pasternak-type foundation. The results showed that the nonlinearity can be tuned by applying axial tension to the nanotube. Khosrozadeh and Hajabasi [7] used the harmonic balance method for nonlinear free vibration of DWCNTs based on Euler–Bernoulli beam theory modeled by van der Waals force. The same procedure was applied by Fang et al. [8] for free vibration of clamped–clamped DWCNTs taking into account von Karman geometric nonlinearity, showing that the amplitudes of vibration in the case of van der Waals forces are larger than those considering both geometric nonlinearity and van der Waals force. Arani et al. [9] investigated single-walled boron nitride nanotubes (SWBNNTs) by means of the method of multiple scales and of differential quadrature. The interaction between the inner viscous fluid and SWBNNTs was studied by means of Navier–Stokes equations decoupling the electrical and mechanical fields. The parametrized perturbation method was proposed by Valipour et al. [10] to characterize the nonlinear vibration of embedded SWCNTs conveying fluid, employing Pasternak-type elastic foundation. The result is that by increasing the Winkler constant, the nonlinear frequency decreases. A modified variational iteration method was proposed by Pashaki and Li [11] to study strongly nonlinear vibration of CNTs conveying viscous fluid, which is a damped vibrational system. The results showed that with an increase in amplitude and incorporating the geometric nonlinearity, the effect of the viscosity became more apparent. Miashiro et al. [12] explored the mechanical vibration of SWCNTs at different lengths and carbon nanobelt forms with beam elements, showing that SWCNTs and carbon nanobelts smaller than the aspect ratio 1 significantly increase the static modulus and their first vibration mode also changes from bending mode to the radial mode. By using Fourier-transform infrared spectroscopy, Line et al. [13] employed the impacts of CNTs and titanium dioxide nanoparticles on tomato plants after a number of days of exposure in soil. They established modifications in leaf cell wall components of plants. Civalek et al. [14] analyzed the free vibration behavior of carbon nanotube-reinforced composite microbeams. CNTs are distributed in a polymeric matrix with four patterns of the reinforcement and the material properties are predicted by the rule of mixture T. The free vibration of a tapered beam modeling nonuniform SWCNT i.e., nanocones, was studied by De Rosa et al. [15] using the differential quadrature method applied to compute the natural frequencies for the clamped beam at one end and elastically restrained at the other. Su and Cho [16] examined the influences of the slenderness ratios, the boundary conditions, the atomic structures and the stiffness of the embedded medium on the natural frequencies and mode shapes of SWCNTs on the basis of the nonlocal cantilever Timoshenko beam model. Senthilkumar [17] studied the axial vibration of DWCNTs without decoupling the continuum model by means of the semianalytical procedure. The Pasternak medium support and magnetic effects influence the frequencies of the first and the second nanotubes. Hossain and Lellep [18] presented the Maclaurin series approach to solve the equation of motion derived from the Euler–Bernoulli beam theory based on Eringen’s nonlocal theory of elasticity. Accordingly, the upper beam influences the mode shape of the upper beams as well as the intact lower beam. Chen et al. [19] investigated the forced vibrations of a functionally graded double-nanobeam system on an elastic foundation using Eringen’s nonlocal elasticity theory. The explicit expressions of the stationary responses were obtained by Green’s functions method in conjunction with the superposition principle, leading to the conclusion that the bond between the two nanobeams can be significantly reinforced by increasing the stiffness and damping coefficient of the connecting layer.

Different practical applications of CNTs in various devices and materials or structures were recently reported [20,21,22,23], emphasizing their usefulness and efficiency. Baydin et al. [20] presented a comprehensive review of the current developments in the field of carbon nanotube devices for quantum technology, discussing recent progress in the development of carbon-nanotube-based devices and strategies for revolutionizing computation, sensing, and communication as components of quantum technology. Oh et al. [21] proposed an application of carbon nanotubes (CNTs), a conductive carbon nanofiller, and polydimethylsiloxane as a polymer matrix, to fabricate a flexible pressure sensor, and its piezoresistive and capacitive pressure-sensing characteristics were deeply analyzed, emphasizing a great potential for practical applications in manufacturing pressure sensors. Merodio-Perea et al. [22] studied the mechanical properties of cement reinforced with pristine and functionalized carbon nanotubes, showing that the incorporation of CNTs enhances the mechanical properties of tobermorite as a consequence of better interactions that are established at the cement–CNT interface. Páez-Pavón et al. [23] reported interesting results of the addition of different types of carbon nanotubes on the microstructure and on the physical properties of cement paste. The microstructure was analyzed and the electrical conductivity for different CNT concentrations was measured, identifying improved features of great interest in the field of construction materials.

In the present study, we investigated the nonlinear forced vibration of DWCNTs subjected to mechanical impact and embedded on Winkler–Pasternak foundation in the neighborhood of the primary resonance. Two Euler–Bernoulli beams interacting through van der Waals forces are considered in the paper. Using the Hamilton principle and Eringen’s nonlocal elastic theory, taking into consideration the moving nanoparticles, we obtain two partial differential equations as governing equations, which are reduced by means of the Galerkin–Bubnov procedure to two differential equations with variable coefficients. For the first time, we considered the cumulated effects of nonlinearities induced by the Winkler–Pasternak foundation, the curvature of beams and the nonlinear van der Waals force, and also the effect of discontinuities marked by the presence of the Dirac function. The nonlinear differential equations are studied by means of the optimal auxiliary functions method (OAFM). An explicit and very accurate analytical approximate solution is obtained using the so-called auxiliary functions and a moderate number of convergence-control parameters, independently of the presence of small or large parameters in the governing equations or in the boundary/initial conditions. Finally, a stability analysis of the considered model is developed by means of the homotopy perturbation method (HPM) using the condition of existence of the two frequencies, which led to the establishment of the borderline cases, all these being of much help in guiding the design of advanced nanoelectromechanical devices, in which nanotubes act as basic elements.

## 2. Governing Equations

**Figure 1:**
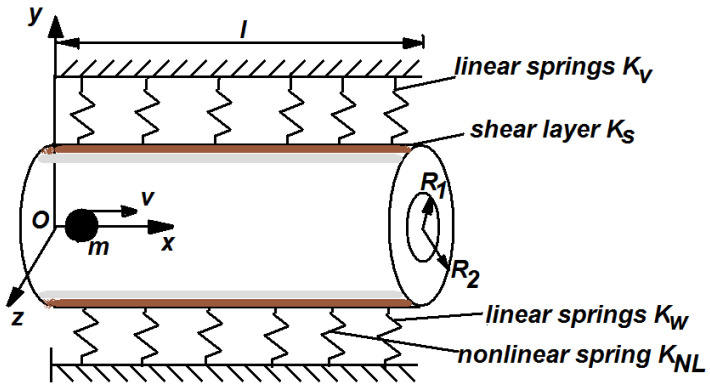
Geometry of DWCNT on Winkler–Pasternak foundation under the moving nanoparticle.

In Figure 1 is depicted a double tube of length *l*, having inner radius R_1_, outer radius R_2_, Young’s modulus E and density ρ.

The Winkler–Pasternak foundation is characterized by shear layer constant K_s_, the linear spring K_w_ and nonlinear spring K_NL_. Through inner tube moves the nanoparticle of mass m and with constant speed v. While the nanoparticle arrives at the right end, another nanoparticle moves immediately. Simply supported boundary conditions are assumed. The extended Hamilton principle for a system with changing mass and based on Euler–Bernoulli beam theory can be written as

(1) $$\int_{t_{1}}^{t_{2}}(\delta T-\delta \Pi +\delta W_{NP})dt=0$$

where *T*, Π and *W_NP_* are kinetic energy, potential energy (which include the strain energy of nanotube, potential energy from Winkler–Pasternak foundation and potential energy due to van der Waals forces) and the work done by the moving nanoparticle m, respectively. The Euler–Bernoulli theory supposes that the vector perpendicular to the neutral axis x of the beam remains perpendicular after deformation and accordingly, the deformation due to shear stress is neglected. Also, the longitudinal displacement of the beam can be neglected.

The displacement field can be written as [4,5,7,8]:

(2) $$\begin{array}{l} {\bar{U}}_{i}(x,z,t)=-z\frac{\partial W_{i}(x,t)}{\alpha x}\\ {\bar{V}}_{i}(x,z,t)=0\\ {\bar{W}}_{i}(x,z,t)=W_{i}(x,t)\begin{array}{cc} , & i=1,2 \end{array} \end{array}$$

where ${\bar{U}}_{i}$, ${\bar{V}}_{i}$, ${\bar{W}}_{i}$ are the displacements of an arbitrary point along *x*-, *y*-, *z*- directions, respectively. For small deflection, the strain component is

(3) $$\varepsilon_{xx}=-z\frac{\partial^{2}W_{i}}{\partial x^{2}}$$

Hooke’s law for linear deformations becomes:

(4) $$\sigma_{xx}=E\varepsilon_{xx}$$

The kinetic energy of DWCNT is

(5) $$T=\frac{1}{2}\rho \int_{0}^{l}\left(\int_{A_{1}}{\left(\frac{\partial W_{1}}{\partial t}\right)}^{2}dA_{1}\right)dx+\frac{1}{2}\rho \int_{0}^{l}\left(\int_{A_{2}}{\left(\frac{\partial W_{2}}{\partial t}\right)}^{2}dA_{2}\right)dx$$

where *A*_1_ and *A*_2_ are cross-sections of the inner and outer tubes, respectively.

The strain energy of nanotubes stored in the DWCNT is

(6) $$\Pi_{s}=\frac{1}{2}E\int_{0}^{l}\left(\int_{A_{1}}{\left(z\frac{\partial^{2}W_{1}}{\partial x^{2}}\right)}^{2}dA_{1}\right)dx+\frac{1}{2}E\int_{0}^{l}\left(\int_{A_{2}}{\left(z\frac{\partial^{2}W_{2}}{\partial x^{2}}\right)}^{2}dA_{2}\right)dx$$

The potential energy derived from Winkler–Pasternak foundation can be written in the form

(7) $$\Pi_{WP}=\frac{1}{2}\int_{0}^{l}\left(K_{W}W_{2}+K_{NL}W_{2}^{3}-K_{s}\frac{\partial^{2}W_{2}}{\partial x^{2}}\right)W_{2}dx$$

The potential energy derived from van der Waals forces can be given by

(8) $$\Pi_{v}=-\frac{1}{2}\int_{0}^{l}P_{12}W_{1}dx-\frac{1}{2}\int_{0}^{l}P_{21}W_{2}dx$$

where *P*_12_ is the force applied to the outer tube by the inner one and *P*_21_ is the force applied to the inner tube by the outer one. In this case it holds that *P*_12_ = −*P*_21_ = *P_v_* and for the nonlinear analyses the potential of van der Waals forces can be extended by Taylor expansion around the equilibrium points as [9]:

(9) $$\Pi_{v}=C_{1}(W_{2}-W_{1})+C_{3}{\left(W_{2}-W_{1}\right)}^{3}$$

The work done by the moving particle *m* is

(10) $$W_{NP}=-\int_{0}^{l}F(x,t)W_{1}dx$$

in which *F*(*x*,*t*) is a combination of the loading function with the inertial effect of the moving nanoparticle

(11) $$F(x,t)=\left[m\left(g-\frac{D^{2}W_{1}}{Dt^{2}}\right)-K_{v}W_{1}\right]H(l-x)\delta (x-Vt)$$

where the material derivative of *W*_1_ can be expressed as

(12) $$\frac{D^{2}W_{1}}{Dt^{2}}=\frac{\partial^{2}W_{1}}{\partial x^{2}}+2V\frac{\partial^{2}W_{1}}{\partial x\partial t}+V^{2}\frac{\partial^{2}W_{1}}{\partial t^{2}}$$

and *g* is the gravity acceleration, *K_v_* is the linear spring constant utilized to simulate the van der Waals force between the mass *m* and the DWCNT, *H* is the Heaviside function and *δ* is the Dirac function.

Substituting Equations (11) and (12) into Equation (10), we have:

(13) $$W_{NP}==\int_{0}^{l}\left[m\left(g-\frac{\partial^{2}W_{1}}{\partial x^{2}}-2V\frac{\partial^{2}W_{1}}{\partial x\partial t}-V^{2}\frac{\partial^{2}W_{1}}{\partial t^{2}}\right)-K_{v}W_{1}\right]H(l-x)\delta (x-vt)dx$$

Substituting Equations (5)–(8) and (13) into Equation (1) and calculating the variation of *W*_1_ and *W*_2_, the governing equations of motion are:

(14) $$\rho A_{1}\frac{\partial^{2}W_{1}}{\partial t^{2}}-\frac{\partial^{2}M_{1}}{\partial x^{2}}-\left[m\left(g-\frac{\partial^{2}W_{1}}{\partial x^{2}}-2V\frac{\partial^{2}W_{1}}{\partial x\partial t}-V^{2}\frac{\partial^{2}W_{1}}{\partial t^{2}}\right)-K_{v}W_{1}\right]H(l-x)\delta (x-vt)-C_{1}(W_{2}-W_{1})-C_{3}{\left(W_{2}-W_{1}\right)}^{3}=0$$

(15) $$\rho A_{2}\frac{\partial^{2}W_{2}}{\partial t^{2}}-\frac{\partial^{2}M_{2}}{\partial x^{2}}+(K_{W}W_{2}+K_{NL}W_{2}^{3}-K_{s}\frac{\partial^{2}W_{2}}{\partial t^{2}})+C_{1}(W_{2}-W_{1})+C_{3}{\left(W_{2}-W_{1}\right)}^{3}=0$$

Based on Eringen’s nonlocal theory [24], the constitutive relations for homogeneous isotropic beams can be approximated by using Green’s function as [5]:

(16) $$\sigma_{xx}-\mu^{2}\frac{\partial^{2}\sigma_{xx}}{\partial x^{2}}=E\varepsilon_{xx}$$

From the last equation, after it is multiplied by z and then is integrated, the nonlocal stress resultant becomes:

(17) $$M_{i}-\mu^{2}\frac{\partial^{2}M_{i}}{\partial x^{2}}=-EI\frac{\partial^{2}W_{i}}{\partial x^{2}}$$

where we used the relation

(18) $$\int_{A_{i}}zdA_{i}=0\begin{array}{cc} , & i=1,2 \end{array}$$

Using the approximation

(19) $$\frac{\frac{\partial^{2}W_{i}}{\partial x^{2}}}{\sqrt{1+{\left(\frac{\partial W_{i}}{\partial x}\right)}^{2}}}=\frac{\partial^{2}W_{i}}{\partial x^{2}}\left[1-\frac{3}{2}{\left(\frac{\partial W_{i}}{\partial x}\right)}^{2}\right]$$

and performing some manipulations, from Equations (14), (15), (17) and (19), the governing equations of motion are:

(20) $$\begin{array}{l} \rho A_{1}\frac{\partial^{2}W_{1}}{\partial t^{2}}-\mu^{2}\rho A_{1}\frac{\partial^{4}W_{1}}{\partial x^{2}\partial t^{2}}+EI_{1}\left[\frac{\partial^{4}W_{1}}{\partial x^{4}}-\frac{3}{2}\frac{\partial^{4}W_{1}}{\partial x^{4}}{\left(\frac{\partial W_{1}}{\partial x}\right)}^{2}-9\frac{\partial W_{1}}{\partial x}\frac{\partial^{2}W_{1}}{\partial x^{2}}\frac{\partial^{3}W_{1}}{\partial x^{3}}-3{\left(\frac{\partial^{2}W_{1}}{\partial x^{2}}\right)}^{3}\right]-[m(g-\frac{\partial^{2}W_{1}}{\partial x^{2}}-2V\frac{\partial^{2}W_{1}}{\partial x\partial t}-\\ -V^{2}\frac{\partial^{2}W_{1}}{\partial t^{2}}-K_{v}W_{1}]H(l-x)\delta (x-vt)-C_{1}(W_{2}-W_{1})-C_{3}{\left(W_{2}-W_{1}\right)}^{3}+\mu^{2}C_{1}\left(\frac{\partial^{2}W_{2}}{\partial x^{2}}-\frac{\partial^{2}W_{1}}{\partial x^{2}}\right)+\mu^{2}C_{3}\frac{\partial^{2}}{\partial x^{2}}{\left(W_{2}-W_{1}\right)}^{3}=0 \end{array}$$

(21) $$\begin{array}{l} \rho A_{2}\frac{\partial^{2}W_{2}}{\partial t^{2}}-\mu^{2}\rho A_{2}\frac{\partial^{4}W_{2}}{\partial x^{2}\partial t^{2}}+EI_{2}\left[\frac{\partial^{4}W_{2}}{\partial x^{4}}-\frac{3}{2}\frac{\partial^{4}W_{2}}{\partial x^{4}}{\left(\frac{\partial W_{2}}{\partial x}\right)}^{2}-9\frac{\partial W_{2}}{\partial x}\frac{\partial^{2}W_{2}}{\partial x^{2}}\frac{\partial^{3}W_{2}}{\partial x^{3}}-3{\left(\frac{\partial^{2}W_{1}}{\partial x^{2}}\right)}^{3}\right]+K_{W}W_{2}+K_{NL}W_{2}^{3}-\\ -K_{s}\frac{\partial^{2}W_{2}}{\partial t^{2}}-\mu^{2}K_{W}\frac{\partial^{2}W_{2}}{\partial x^{2}}-\mu^{2}K_{NL}\frac{\partial^{2}W_{2}}{\partial x^{2}}+\mu^{2}K_{s}\frac{\partial^{4}W_{2}}{\partial x^{4}}+C_{1}(W_{2}-W_{1})+C_{3}{\left(W_{2}-W_{1}\right)}^{3}-\\ -\mu^{2}C_{1}\left(\frac{\partial^{2}W_{2}}{\partial x^{2}}-\frac{\partial^{2}W_{1}}{\partial x^{2}}\right)-\mu^{2}C_{3}\frac{\partial^{2}}{\partial x^{2}}{\left(W_{2}-W_{1}\right)}^{3}=0 \end{array}$$

In order to discretize the Equations (20) and (21), by applying the Galerkin–Bubnov procedure, the displacement of the DWCNT for the simply supported case are given by

(22) $$\begin{array}{l} W_{1}(x,t)=\sqrt{\frac{2}{l}}\left(\sin\frac{\pi x}{l}\right)T_{1}(t)\\ W_{2}(x,t)=\sqrt{\frac{2}{l}}\left(\sin\frac{\pi x}{l}\right)T_{2}(t) \end{array}$$

Substituting Equation (22) into Equations (20) and (21), multiplying then by $\sqrt{\frac{2}{l}}\sin\frac{\pi x}{l}$ and integrating over the domain [0,*l*], one gets:

(23) $$\begin{array}{l} \left[\rho A_{1}+\mu^{2}\rho A_{1}\frac{\pi^{2}}{l^{2}}+2\frac{m}{l}\sin^{2}\left(\frac{\pi vt}{l}\right)+2\mu^{2}\frac{\pi^{2}}{l^{3}}\sin^{2}\left(\frac{\pi vt}{l}\right)\right]{\ddot{T}}_{1}+[4mv\frac{\pi }{l^{2}}\sin\left(\frac{\pi vt}{l}\right)\cos\left(\frac{\pi vt}{l}\right)+4mv^{2}\frac{\pi^{3}}{l^{4}}\sin\left(\frac{\pi vt}{l}\right)\cos\left(\frac{\pi vt}{l}\right)]{\dot{T}}_{1}+\\ +[EI_{1}\frac{\pi^{4}}{l^{4}}-2mv^{2}\frac{\pi^{2}}{l^{3}}\sin^{2}\left(\frac{\pi vt}{l}\right)-2m\mu^{2}v^{2}\frac{\pi^{4}}{l^{3}}\sin^{2}\left(\frac{\pi vt}{l}\right)+\frac{2}{l}K_{v}\sin^{2}\left(\frac{\pi vt}{l}\right)+2\mu^{2}\frac{K_{v}\pi^{2}}{l^{3}}\sin^{2}\left(\frac{\pi vt}{l}\right)+C_{1}+\mu^{2}C_{1}\frac{\pi^{2}}{l^{2}}]T_{1}-\\ -C_{1}(1+\mu^{2}\frac{\pi^{2}}{l^{2}})T_{2}-EI_{1}\frac{3l}{16}{\left(\frac{\pi }{l}\right)}^{6}{\left(\sqrt{\frac{2}{l}}\right)}^{3}T_{1}^{3}-\frac{3C_{3}}{4}\sqrt{\frac{2}{l}}\left(\frac{\pi^{2}\mu^{2}}{l^{2}}-1\right){\left(T_{2}-T_{1}\right)}^{3}=mg\sqrt{\frac{2}{l}}\sin\left(\frac{\pi vt}{l}\right) \end{array}$$

(24) $$\begin{array}{l} \left[\rho A_{2}+\mu^{2}\rho A_{2}\frac{\pi^{2}}{l^{2}}\right]{\ddot{T}}_{2}+[EI_{2}\frac{\pi^{4}}{l^{4}}+K_{v}+\mu^{2}K_{W}\frac{\pi^{2}}{l^{2}}+K_{s}\frac{\pi^{2}}{l^{2}}+\mu^{2}K_{s}\frac{\pi^{4}}{l^{2}}+C_{1}+C_{1}\mu^{2}\frac{\pi^{2}}{l^{2}}]T_{2}-\\ -EI_{2}\frac{3l}{16}{\left(\frac{\pi }{l}\right)}^{6}{\left(\sqrt{\frac{2}{l}}\right)}^{3}T_{2}^{3}+\frac{3C_{3}}{4}\sqrt{\frac{2}{l}}\left(\frac{\pi^{2}\mu^{2}}{l^{2}}-1\right){\left(T_{2}-T_{1}\right)}^{3}-K_{NL}{\left(\sqrt{\frac{2}{l}}\right)}^{3}\frac{3l}{8}(1+\mu^{2}\frac{\pi^{2}}{l^{2}})T_{2}^{3}-C_{1}(1+\mu^{2}\frac{\pi^{2}}{l^{2}})T_{1}=0 \end{array}$$

By means of non-dimensional parameters

(25) $$\begin{array}{l} \tau *=\frac{\pi vt}{l};\;\;\xi =\frac{m}{\rho A_{1}l};\;\;Q_{i}=EI_{i}{\left(\frac{\pi }{l}\right)}^{4}T_{i};\;\;{\bar{d}}_{1}=\frac{\pi^{2}EI_{1}}{\rho A_{1}{\left(vl\right)}^{2}\left(1+\frac{\mu^{2}\pi^{2}}{l^{2}}\right)}+\frac{l^{2}C_{1}}{\pi^{2}v^{2}\rho A_{1}}+\frac{K_{v}l}{\pi^{2}v^{2}\rho A_{1}}-\frac{m}{\rho A_{1}l};\;\;{\dot{d}}_{2}=\frac{K_{v}l}{\rho A_{1}A^{2}\pi^{2}}-\frac{m}{\rho A_{1}l}\\ e_{1}=\frac{C_{1}l^{2}}{\pi^{2}v^{2}\rho A_{1}};\;\;{\bar{a}}_{1}=\frac{3\sqrt{2}}{8}\frac{\pi^{2}}{l^{2}\left(1+\frac{\mu^{2}\pi^{2}}{l^{2}}\right)};\;\;{\bar{b}}_{1}=\frac{3C_{3}}{4EI_{1}}\sqrt{\frac{2}{l}}{\left(\frac{l}{\pi }\right)}^{4};\;\;\bar{f}=\frac{\sqrt{2}gl}{\pi^{2}v^{2}\left(1+\frac{\mu^{2}\pi^{2}}{l^{2}}\right)}\;\;{\bar{b}}_{2}=-\frac{3C_{3}}{4EI_{2}}\sqrt{\frac{2}{l}}{\left(\frac{l}{\pi }\right)}^{4}\\ {\bar{d}}_{3}=\frac{\pi^{2}EI_{2}}{\rho A_{2}{\left(vl\right)}^{2}\left(1+\frac{\mu^{2}\pi^{2}}{l^{2}}\right)}+\frac{l^{2}C_{1}}{\pi^{2}v^{2}\rho A_{2}}+\frac{K_{s}+K_{W}}{\pi^{2}v^{2}\rho A_{2}};\;\;e_{2}=\frac{C_{1}l^{2}}{\pi v^{2}\rho A_{2}};\;\;{\bar{a}}_{2}=\frac{3\sqrt{2}}{8}\frac{\pi^{2}}{l^{2}\left(1+\frac{\mu^{2}\pi^{2}}{l^{2}}\right)}+\frac{K_{NL}EI_{2}}{\rho A_{2}}\frac{3\sqrt{2}\pi^{2}}{8v^{2}l} \end{array}$$

the governing Equations (23) and (24) can be written as

(26) $$\left[1+\xi (1-\cos2\tau *)\right]Q_{1}^{\prime \prime }+2(\sin2\tau *)Q_{1}^{\prime }+({\bar{d}}_{1}-{\bar{d}}_{2}\cos2\tau *)Q_{1}-{\bar{e}}_{1}Q_{2}-{\bar{a}}_{1}Q_{1}^{3}-{\bar{b}}_{1}{\left(Q_{2}-Q_{1}\right)}^{3}=\bar{f}\sin\tau *$$

(27) $$Q_{2}^{\prime \prime }+d_{3}Q_{2}-{\bar{e}}_{2}Q_{1}-{\bar{a}}_{2}Q_{2}^{3}+{\bar{b}}_{2}{\left(Q_{2}-Q_{1}\right)}^{3}=0$$

where the prime denotes differentiation with respect to τ*.

The initial conditions for Equations (26) and (27) are

(28) $$Q_{1}(0)=A\begin{array}{cc} ; & Q_{1}^{\prime }(0)=0 \end{array}\begin{array}{cc} ; & Q_{2}(0)=B \end{array}\begin{array}{cc} ; & Q_{2}^{\prime }(0)=0 \end{array}$$

Equations (26)–(28) are second-order nonlinear differential equations with variable coefficients, and therefore are very difficult to be analytically solved. In what follows, for Equations (26)–(28), the OAFM is applied to study the nonlinear vibration near the primary resonance.

## 3. Basics of the Optimal Auxiliary Functions Methods

For a general nonlinear differential equation [25,26,27,28,29,30,31]

(29) $$L\left[Q\left(\tau \right)\right]+N\left[Q\left(\tau \right)\right]=0\begin{array}{cc} , & \tau \in D \end{array}$$

with the initial conditions

(30) $$B\left[Q\left(\tau \right),\frac{dQ\left(\tau \right)}{d\tau }\right]=0$$

where *L* is a linear operator, *N* is a nonlinear operator, *D* is the domain of interest, *B* is a boundary operator, we propose that the approximate solution $\bar{Q}\left(\tau \right)$ to be of the form

(31) $$\bar{Q}\left(\tau \right)=Q_{0}\left(\tau \right)+Q_{1}(\tau ,C_{1},C_{2},\ldots ,C_{n})$$

where *C_i_* are n parameters unknown at this moment and n is an arbitrary positive integer number. The initial approximation *Q*_0_(τ) can be determined from the linear differential equation

(32) $$L\left[Q_{0}\left(\tau \right)\right]=0$$

with the initial conditions

(33) $$B\left[Q_{0}\left(\tau \right),\frac{dQ_{0}\left(\tau \right)}{d\tau }\right]=0$$

Taking into consideration the last two equations, the first approximation *Q*_1_(τ,*C_i_*) is obtained from the nonlinear differential equation

(34) $$L\left[Q_{1}\left(\tau ,C_{i}\right)\right]+N\left[Q_{0}\left(\tau \right)+Q_{1}\left(\tau ,C_{i}\right)\right]=0$$

with the initial conditions

(35) $$B\left[Q_{1}\left(\tau ,C_{i}\right),\frac{dQ_{1}\left(\tau ,C_{i}\right)}{d\tau }\right]=0$$

However, the nonlinear differential Equations (34) and (35) are very difficult to solve. We propose the following alternative. The nonlinear term of Equation (34) is developed in the form

(36) $$N[Q_{0}(\tau )+Q_{1}\left(\tau ,C_{i}\right)]=N[Q_{0}(\tau )]+\sum_{k\geq 1}\frac{Q_{1}^{k}\left(\tau ,C_{i}\right)}{k!}N^{\left(k\right)}[Q_{0}(\tau )]$$

where *k*! = 1, 2, …, *k* and *N*^(*k*)^ denotes differentiation of order *k* of the nonlinear operator *N*. To avoid the difficulties that appear in solving the nonlinear differential Equation (36), and to accelerate the convergence of the first approximate solution *Q*_1_, instead of solving the following equation obtained from Equations (34) and (36):

(37) $$L[Q_{1}\left(\tau ,C_{i}\right)]+N[Q_{0}(\tau )+\sum_{k\geq 1}\frac{Q_{1}^{k}\left(\tau ,C_{i}\right)}{k!}N^{\left(k\right)}[Q_{0}(\tau )]=0$$

we make the fundamental remarks as follows. In general, the solution of liner differential Equations (32) and (33) is known and can be expressed in the form

(38) $$Q_{0}(\tau )=\sum_{i=1}^{p}a_{i}f_{i}(\tau )$$

where the coefficients a_i_, the functions *f_i_*(τ) and integer positive *p* are known. Now, the nonlinear operator *N*[*Q*_0_(τ)] calculated for *Q*_0_(τ) given by Equation (38), may be written as

(39) $$N[Q_{0}(\tau )]=\sum_{j=1}^{q}b_{j}g_{j}(\tau )$$

where the coefficients *b_j_*, the functions *g_i_*(τ) and positive integer *q* are known and depend on the initial approximation *Q*_0_(τ) and also on the nonlinear operator *N*. Since Equation (37) is very difficult to solve, according to OAFM procedure, it is more convenient to consider that the first approximation *Q*_1_(τ,*C_i_*) can be solution of linear differential equation

(40) $$L[Q_{1}\left(\tau ,C_{i}\right)]=\sum_{j=k}^{k+m}H_{j}(\tau ,C_{1},C_{2},\ldots ,C_{n})g_{i}\begin{array}{cc} , & B\left(Q_{1}\left(\tau ,C_{i}\right),\frac{dQ_{1}\left(\tau ,C_{i}\right)}{d\tau }\right) \end{array}=0$$

where *H_j_* are *m* < *p* so-called auxiliary functions which depend on n parameters C_i_ and can be chosen as mathematical expressions similar with *Q*_0_(τ) and *N*[*Q*_0_(τ)]. The functions g_j_ are only m functions involved in Equation (39). In the case of vibration problems, the resonant terms into Equation (40) must be avoided. From this condition, we can obtain the expression of the frequency. After this clarification, the linear Equation (40) can be easily solved.

In consequence, the first approximation is determined from Equation (40) and the approximate solution of Equations (29) and (30) is determined from Equations (31), (32) and (40).

Finally, the unknown parameters *C_i_*, *i* = 1, 2, …, *n* can be optimally identified via rigorous mathematical procedures, such as the Galerkin method, least square method, Ritz method, collocation method, Kantorovich method, and so on.

In this way, the optimal values of the convergence-control parameters are known and accordingly, the approximate solution $\bar{Q}(\tau )$ is well-determined. Let us note that the nonlinear differential Equations (29) and (30) are reduced to two linear differential equations. This technique led to a very accurate result, is effective, explicit and provides a rigorous way to control and adjust the convergence of the solutions, without the presence of small parameters.

## 4. Application of OAFM to the Governing Equations of Nonlinear DWCNT System

If ω_1_ and ω_2_ are the frequencies of the tubes, and taking into consideration initial conditions (28), we make the following transformations:

(41) $$\begin{array}{lllllll} Q_{1}=XA, & Q_{2}=YB, & \tau_{1}=2\omega_{1}\tau *, & \tau_{2}=2\omega_{2}\tau *, & a_{1}=\frac{{\bar{a}}_{1}}{A^{2}}, & b_{1}=\frac{{\bar{b}}_{1}}{B^{2}}, & a_{2}=\frac{{\bar{a}}_{2}}{B^{2}}\\ b_{2}=\frac{{\bar{b}}_{2}A^{3}}{B}, & f=\frac{\bar{f}}{A}, & d_{1}=\frac{{\bar{d}}_{1}}{A^{2}}, & d_{2}=\frac{{\bar{d}}_{2}}{A^{2}}, & d_{3}=\frac{{\bar{d}}_{3}}{B^{2}}, & e_{1}=\frac{{\bar{e}}_{1}B}{A}, & e_{2}=\frac{{\vec{e}}_{2}A}{B} \end{array}$$

such that Equations (26) and (27) can be rewritten as

(42) $$X^{\prime \prime }+X+\xi (1-\cos\frac{\tau_{1}}{\omega_{1}})X^{\prime \prime }+\frac{\xi }{2\omega_{1}}(\sin\frac{\tau_{1}}{\omega_{1}})X^{\prime }+\left(\frac{d_{1}}{4\omega_{1}}-\frac{d_{2}}{4\omega_{1}}\cos\frac{\tau_{1}}{\omega_{1}}\right)X-\frac{e_{1}}{4\omega_{1}^{2}}Y-\frac{a_{1}X^{3}}{4\omega_{1}^{2}}-\frac{b_{1}}{4\omega_{1}^{2}}{\left(\frac{B}{A}Y-X\right)}^{3}=\frac{f}{4\omega_{1}^{2}}\sin\frac{\tau_{1}}{2\omega_{1}}$$

(43) $$Y^{\prime \prime }+Y+\left(\frac{d_{3}}{4\omega_{2}^{2}}-1\right)Y-\frac{e_{2}}{4\omega_{2}^{2}}X-\frac{a_{2}}{4\omega_{2}^{2}}Y^{3}+\frac{b_{2}}{4\omega_{2}^{2}}{\left(\frac{B}{A}Y-X\right)}^{3}=0$$

The linear and nonlinear operators corresponding to Equations (42) and (43) are respectively

(44) $$\begin{array}{l} L[X(\tau_{1})]=X^{\prime \prime }+X\begin{array}{cc} , & \end{array}N[X(\tau_{1})]=\xi (1-\cos\frac{\tau_{1}}{\omega_{1}})X^{\prime \prime }+\frac{\xi }{2\omega_{1}}(\sin\frac{\tau_{1}}{\omega_{1}})X^{\prime }+\\ +\left(\frac{d_{1}}{4\omega_{1}^{2}}-\frac{d_{2}}{4\omega_{1}^{2}}\cos\frac{\tau_{1}}{\omega_{1}}\right)X-\frac{e_{1}}{4\omega_{1}^{2}}Y-\frac{a_{1}}{4\omega_{1}^{2}}X^{3}-\frac{b_{1}}{4\omega_{1}^{2}}{\left(\frac{B}{A}Y-X\right)}^{3}-\frac{f}{4\omega_{1}^{2}}\sin\frac{\tau_{1}}{2\omega_{1}} \end{array}$$

(45) $$L[Y(\tau_{2})]=Y^{\prime \prime }+Y\begin{array}{cc} , & \end{array}N[Y(\tau_{2})]=\left(\frac{d_{3}}{4\omega_{2}^{2}}-1\right)Y-\frac{e_{2}}{4\omega_{2}^{2}}X-\frac{a_{2}}{4\omega_{2}^{2}}Y^{3}+\frac{b_{2}}{4\omega_{2}^{2}}{\left(\frac{B}{A}Y-X\right)}^{3}$$

The initial conditions for nonlinear Equations (42) and (43) are

(46) $$X(0)=1\begin{array}{cc} , & X^{\prime }(0)=0 \end{array}\begin{array}{cc} , & Y(0)=1 \end{array}\begin{array}{cc} , & Y^{\prime }(0)=0 \end{array}$$

The approximate solutions of Equations (42) and (43) can be written as

(47) $$\bar{X}(\tau_{1})=X_{0}(\tau_{1})+X_{1}(\tau_{1})\begin{array}{cc} ; & \bar{Y}(\tau_{2}) \end{array}=Y_{0}(\tau_{2})+Y_{1}(\tau_{2})$$

The initial approximations *X*_0_(τ_1_) and *Y*_0_(τ_2_) are determined from the following linear differential equations

(48) $$L[X_{0}(\tau_{1})]=0\begin{array}{cc} , & L[ \end{array}Y_{0}(\tau_{2})]=0\begin{array}{cc} , & X_{0}(0)=Y_{0}(0)=1\begin{array}{cc} , & X_{0}^{\prime }(0)=Y_{0}^{\prime }(0)=0 \end{array} \end{array}$$

whose solutions are, respectively,

(49) $$X_{0}(\tau_{1})=\cos\tau_{1}\begin{array}{cc} , & Y_{0}(\tau_{2})=\cos\tau_{2} \end{array}$$

in which ω_1_ and ω_2_ are unknown.

Inserting Equation (48) into the second equations of (43) and (44), we obtain

(50) $$\begin{array}{l} N[X_{0}(\tau )]=\left(-\xi +\frac{d_{1}}{4\omega_{1}^{2}}-\frac{3a_{1}}{16\omega_{1}^{2}}+\frac{3b_{1}}{16\omega_{1}^{2}}\right)\cos\tau_{1}+\frac{\xi }{2}\left(\cos\frac{1+\omega_{1}}{\omega_{1}}\tau_{1}+\cos\frac{\omega_{1}-1}{\omega_{1}}\tau_{1}\right)-\frac{\xi }{8\omega_{1}^{2}}\left(\cos\frac{\omega_{1}+1}{\omega_{1}}\tau_{1}+\cos\frac{\omega_{1}-1}{\omega_{1}}\tau_{1}\right)-\\ -\frac{e_{2}}{4\omega_{1}^{2}}\cos\tau_{2}-\frac{a_{1}\cos3\tau_{1}}{16\omega_{1}^{2}}-\frac{b_{1}}{4\omega_{1}^{2}}\left(\frac{\cos3\tau_{2}+3\cos\tau_{1}-\cos3\tau_{1}}{4}-3\cos\tau_{1}\cos3\tau_{2}+3\cos2\tau_{1}\cos\tau_{2}-\frac{3}{2}\cos\tau_{2}\right) \end{array}$$

(51) $$\begin{array}{l} N[Y_{0}(\tau_{2})]=\left(\frac{d_{3}}{4\omega_{2}^{2}}-1-\frac{3a_{2}}{16\omega_{2}^{2}}+\frac{9b_{2}}{4\omega_{2}^{2}}\right)\cos\tau_{2}+\left(\frac{b_{2}-a_{2}}{16\omega_{2}^{2}}\right)\cos3\tau_{2}-\frac{3b_{2}}{8\omega_{2}^{2}}\cos\tau_{1}+\\ +\frac{3b_{2}}{16\omega_{2}^{2}}(2\cos2\tau_{1}\cos\tau_{2}-2\cos\tau_{1}\cos2\tau_{2}-\cos3\tau_{1}-3\cos\tau_{1}) \end{array}$$

The auxiliary functions given in Equation (39) corresponding to Equation (50) can be chosen in many forms, such as

(52) $$H_{1}(\tau_{1})=C_{1}+2C_{2}\cos2\tau_{1}+2C_{3}\cos4\tau_{1}+2C_{4}\cos6\tau_{1}$$

or

(53) $$H_{2}(\tau_{1})=C_{1}+2C_{2}\cos4\tau_{1}+2C_{3}\cos8\tau_{1}$$

or

(54) $$H_{3}(\tau_{1})=C_{1}+2C_{2}\cos4\tau_{1}+2C_{3}\cos6\tau_{1}+2C_{4}\cos8\tau_{1}+2C_{5}\cos10\tau_{1}$$

and so on.

The auxiliary functions for Equation (50) can be:

(55) $$H_{4}(\tau_{2})=C_{5}+2C_{6}\cos2\tau_{2}+2C_{7}\cos4\tau_{2}+2C_{8}\cos6\tau_{2}+2C_{9}\cos8\tau_{2}$$

or

(56) $$H_{5}(\tau_{2})=C_{5}+2C_{6}\cos4\tau_{2}$$

or

(57) $$H_{6}(\tau_{2})=C_{5}+2C_{6}\cos2\tau_{2}+2C_{7}\cos6\tau_{2}$$

Having in view only Equations (51) and (54), the first approximations can be obtained from the linear equations

(58) $$X_{1}^{\prime \prime }+X_{1}=(C_{1}+2C_{2}\cos2\tau_{1}+2C_{3}\cos4\tau_{1}+2C_{4}\cos6\tau_{1})\left[\left(-\xi +\frac{d_{1}}{4\omega_{1}^{2}}-1+\frac{3(b_{1}-a_{1})}{16\omega_{1}^{2}}\right)\cos\tau_{1}+\frac{\xi }{2}\cos\frac{\omega_{1}+1}{\omega_{1}}\tau_{1}\right]$$

(59) $$Y_{1}^{\prime \prime }+Y_{1}=(C_{5}+2C_{6}\cos2\tau_{2}+2C_{7}\cos4\tau_{2}+2C_{8}\cos6\tau_{2})\left[\left(\frac{d_{3}}{4\omega_{2}^{2}}-1-\frac{3a_{2}}{16\omega_{2}^{2}}+\frac{9b_{2}}{4\omega_{2}^{2}}\right)\cos\tau_{2}+\frac{b_{2}-a_{2}}{16\omega_{2}^{2}}\cos3\tau_{2}\right]$$

The secular terms that appear in Equations (57) and (58) have to be canceled by equating the coefficients to zero. This leads to the equations:

(60) $$(C_{1}+C_{2})\left[-\xi +\frac{d_{1}}{4\omega_{1}^{2}}-1+\frac{3(b_{1}-a_{1})}{16\omega_{1}^{2}}\right]-\frac{\left(a_{1}+3b_{1})(C_{2}+C_{3}\right)}{16\omega_{1}^{2}}=0$$

(61) $$(C_{5}+C_{6})\left(\frac{d_{3}}{4\omega_{2}^{2}}-1-\frac{3a_{2}}{16\omega_{2}^{2}}+\frac{9b_{2}}{4\omega_{2}^{2}}\right)+C_{7}\frac{4e_{2}+3b_{2}}{16\omega_{2}^{2}}=0$$

and accordingly, one can get for the case of resonance: *d*_1_ ≈ 4, *d*_3_ ≈ 4:

(62) $$\omega_{1}=\sqrt{\frac{1}{1+\xi }+\frac{3(a_{1}-b_{1})}{16A^{2}(1+\xi )}-\frac{a_{1}+3b_{1}}{16A^{2}(1+\xi )}\frac{C_{2}+C_{3}}{C_{1}+C_{2}}}$$

(63) $$\omega_{2}=\frac{1}{4}\sqrt{16+36b_{2}-a_{2}+\left(4e_{2}+3b_{2}\right)\frac{C_{5}+C_{6}}{C_{7}}}$$

From the initial conditions (47) and (49) it holds that

(64) $$X_{1}(0)=Y_{1}(0)=X_{1}^{\prime }(0)=Y_{1}^{\prime }(0)=0$$

such that

(65) $$X_{1}(\tau_{1})=\frac{C_{1}}{8A}(\cos\tau_{1}-\cos3\tau_{1})+\frac{C_{2}}{24A}(\cos\tau_{1}-\cos5\tau_{1})+\frac{C_{3}}{48A}(\cos\tau_{1}-\cos7\tau_{1})+\frac{C_{4}}{80A}(\cos\tau_{1}-\cos9\tau_{1})$$

(66) $$Y_{1}(\tau_{2})=\frac{C_{5}}{8B}(\cos\tau_{2}-\cos3\tau_{2})+\frac{C_{6}}{24B}(\cos\tau_{2}-\cos5\tau_{2})+\frac{C_{7}}{48B}(\cos\tau_{2}-\cos7\tau_{2})+\frac{C_{8}}{80B}(\cos\tau_{2}-\cos9\tau_{2})$$

In this way, the approximate solutions of Equations (26)–(28), having in view the transformations (41) can be obtained from Equations (46), (48), (63) and (64):

(67) $$\begin{array}{l} {\bar{Q}}_{1}(\tau *)=A\cos2\omega_{1}\tau *+\frac{C_{1}}{8}(\cos2\omega_{1}\tau *-\cos6\omega_{1}\tau *)+\frac{C_{2}}{24}(\cos2\omega_{1}\tau *-\cos10\omega_{1}\tau *)+\\ +\frac{C_{3}}{48}(\cos2\omega_{1}\tau *-\cos14\omega_{1}\tau *)+\frac{C_{4}}{80}(\cos20\omega_{1}\tau *-\cos18\omega_{1}\tau *) \end{array}$$

(68) $$\begin{array}{l} {\bar{Q}}_{2}(\tau *)=B\cos2\omega_{2}\tau *+\frac{C_{5}}{8}(\cos2\omega_{2}\tau *-\cos6\omega_{2}\tau *)+\frac{C_{6}}{24}(\cos2\omega_{2}\tau *-\cos10\omega_{2}\tau *)+\\ +\frac{C_{7}}{48}(\cos2\omega_{2}\tau *-\cos14\omega_{2}\tau *)+\frac{C_{8}}{80}(\cos2\omega_{2}\tau *-\cos18\omega_{2}\tau *) \end{array}$$

## 5. Numerical Example

The efficiency of our procedure can be proved through the following particular case, in which the values of the parameters are

$$A=0.05;B=1;f=0.001;a_{1}=0.002;a_{2}=0.002;b_{1}=0.2;e_{1}=-0.1484344;c_{2}=2.25;b_{2}=0.75;e_{2}=-0.6;\xi =-0.000011168$$

Following the above procedure, the optimal values of the convergence-control parameters are:

$$\begin{array}{l} C_{1}=-0.05860614115;C_{2}=0.00608;C_{3}=-0.0062975641;C_{4}=-0.00290103478;\omega_{1}=1.0542;\omega_{2}=1.044412;\\ C_{5}=-0.035567582192;C_{6}=0.001131969758;C_{7}=-0.000246346225;C_{8}=0.001459685584 \end{array}$$

**Figure 2:**
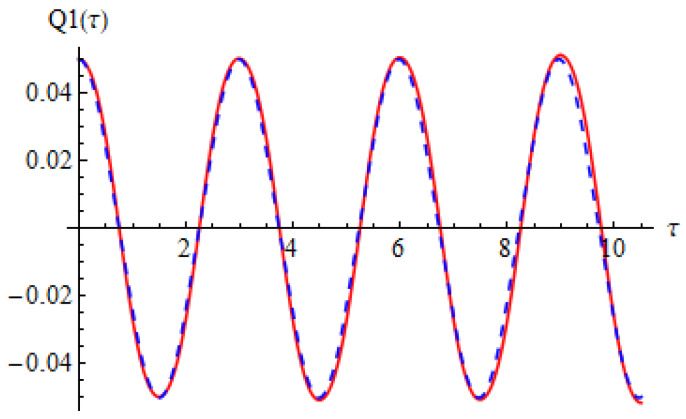
Comparison between the analytical solution (65) and numerical integration results for Equation (26): red solid line depicts numerical integration results while blue dashed line depicts analytical results.

**Figure 3:**
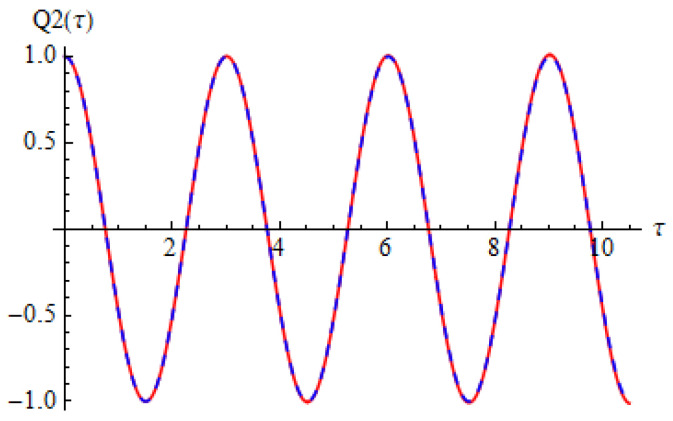
Comparison between the analytical solution (66) and numerical integration results for Equation (27): red solid line depicts numerical integration results while blue dashed line depicts analytical results.

Figure 2 and Figure 3 show the comparison between approximate solution (67) and (68) of nonlinear problems (26)–(28) and numerical solution obtained by means of a fourth-order Runge–Kutta approach.

It can be seen that the two solutions obtained by means of OAFM are nearly identical to the numerical integration results.

## 6. Stability Analysis

The dynamic behavior of the DWCNT system considering mechanical impact and embedded on Winkler–Pasternak foundation is investigated through homotopy perturbation method [32]. This approach predicts limits of instability in the case of primary resonance ${\bar{d}}_{1}\approx 4\begin{array}{cc} , & {\bar{d}}_{3}\approx 4 \end{array}$. The characteristic of this technique is the presence of an embedding parameter *p* ∈ [0,1] such that for p = 0 any nonlinear differential equation reduces to a linear differential equation and for p = 1 one obtains the original equations. The original Equations (26) and (27) are rewritten omitting the bars and stars as:

(69) $$Q_{1}^{\prime \prime }+d_{1}Q_{1}+p\left[\xi (1-\cos2\tau )Q_{1}^{\prime \prime }+2\xi (\sin2\tau )Q_{1}^{\prime }-d_{2}(\cos2\tau )Q_{1}-e_{1}Q_{2}-a_{1}Q_{1}^{3}-b_{1}{\left(Q_{2}-Q_{1}\right)}^{3}-f\sin\tau \right]=0$$

(70) $$Q_{2}^{\prime \prime }+d_{3}Q_{2}+p\left[-e_{2}Q_{1}-a_{2}Q_{2}^{3}+b_{2}{\left(Q_{2}-Q_{1}\right)}^{3}\right]=0$$

We substitute the solution (*Q*_1_, *Q*_2_) and the coefficients *d*_1_ and *d*_3_ by the following power series of *p*:

(71) $$\begin{array}{l} Q_{1}=A_{0}+pA_{1}+p^{2}A_{2}+\ldots \\ Q_{2}=B_{0}+pB_{1}+p^{2}B_{2}+\ldots \\ d_{1}=4=\Omega_{1}^{2}+p\lambda_{1}+p^{2}\lambda_{2}+\ldots \\ d_{3}=4=\Omega_{2}^{2}+p\gamma_{1}+p^{2}\gamma_{2}+,,, \end{array}$$

where *A_i_* and *B_i_* are the solutions of the i-th order homotopy equations, Ω_1_ and Ω_2_ are the response frequencies, λ*_i_* and γ*_i_* are the i-th coefficients of the expansions.

By substituting Equation (71) into the homotopy Equations (69) and (70) and then identifying the coefficients of the same powers, it results a set two linear differential equations. From these linear equations, secular terms are collected and accordingly the values of the coefficients λ*_i_* and γ*_i_* are identified. Each function *A_i_* and *B_i_* are determined separately. Substituting the values of λ*_i_* and γ*_i_* into Equation (71), it suffices to replace *p* = 1 to obtain the frequency Ω_1_ and Ω_2_:

(72) $$\Omega_{1}=\sqrt{4-\lambda_{1}-\lambda_{2}-\ldots }\begin{array}{cc} , & \Omega_{2}=\sqrt{4-\gamma_{1}-\gamma_{2}-\ldots } \end{array}$$

In the case in which the expressions below the radical of Equation (72) become zero, then we can obtain the boundaries curve separating regions of stability and instability.

By means of Equation (71), the Equations (69) and (70) can be rewritten using only three terms as:

(73) $$\begin{array}{l} A_{0}^{\prime \prime }+pA_{1}^{\prime \prime }+p^{2}A_{2}^{\prime \prime }+(\Omega_{1}^{2}+p\lambda_{1}+p^{2}\lambda_{2})(A_{0}+pA_{1}+p^{2}A_{2})+p[\xi (1-\cos2\tau )(A_{0}^{\prime \prime }+pA_{1}^{\prime \prime }+p^{2}A_{2}^{\prime \prime })+2\xi (\sin2\tau )(A_{0}^{\prime }+\\ +pA_{1}^{\prime }+p^{2}A_{2}^{\prime })-d_{2}(\cos2\tau )(A_{0}+pA_{1}+p^{2}A_{2})-e_{1}(B_{0}+pB_{1}+p^{2}B_{2})-a_{1}(A_{0}^{3}+3A_{0}^{2}A_{1}p+(A_{2}^{3}+2A_{0}A_{1})p^{2})-\\ -b_{1}({\left(B_{0}-A_{0}\right)}^{3}+3{\left(B_{0}-A_{0}\right)}^{2}(B_{1}-A_{1})p+({\left(B_{2}-A_{2}\right)}^{2}+2(B_{0}-A_{0})(B_{1}-A_{1}))p^{2}]-f\sin\tau =0 \end{array}$$

(74) $$\begin{array}{l} B_{0}^{\prime \prime }+pB_{1}^{\prime \prime }+p^{2}B_{2}^{\prime \prime }+9\Omega_{2}^{2}+p\gamma_{1}+p^{2}\gamma_{2})(B_{0}+pB_{1}+p^{2}B_{2})+p]-e_{2}(A_{0}+pA_{1}+p^{2}A_{2})-a_{2}(B_{0}^{3}+3B_{0}^{2}B_{1}p+\\ +(B_{2}^{2}+2B_{0}B_{1})p^{2})+b_{2}({\left(B_{0}-A_{0}\right)}^{3}+3{\left(B_{0}-A_{0}\right)}^{2}(B_{1}-A_{1})p+({\left(B_{2}-A_{2}\right)}^{2}+2(B_{0}-A_{0})(B_{1}-A_{1}))p^{2}]=0 \end{array}$$

The initial conditions of Equations (69) and (70) and corresponding Equations (73) and (74) are:

(75) $$Q_{1}(0)=A\begin{array}{cc} , & Q_{1}^{\prime }(0)=0 \end{array}\begin{array}{cc} , & Q_{2}(0)=B \end{array}\begin{array}{cc} , & Q_{2}^{\prime }(0)=0 \end{array}$$

The homotopy equations of order p^i^, i = 0, 1, 2, … and the initial conditions are as follows:

(76) $$\begin{array}{ccc} p^{0}: & & \end{array}A_{0}^{\prime \prime }+\omega_{1}^{2}A_{0}=0\begin{array}{cc} & A_{0}(0)=A \end{array}\begin{array}{cc} , & A_{0}^{\prime }(0)=0 \end{array}$$

(77) $$B_{0}^{\prime \prime }+\omega_{2}^{2}B_{0}=0\begin{array}{cc} & B_{0}(0)=B \end{array}\begin{array}{cc} , & B_{0}^{\prime }(0)=0 \end{array}$$

(78) $$\begin{array}{cc} p^{1}: & \end{array}A_{1}^{\prime \prime }+\Omega_{1}^{2}A_{1}+\lambda_{1}A_{0}+\xi (1-\cos2\tau )A_{0}^{\prime \prime }+2\xi (\sin2\tau )A_{0}^{\prime }-d_{2}(\cos2\tau )A_{0}-e_{1}B_{0}-Q_{1}A_{0}^{3}-b_{1}{\left(B_{0}A_{0}\right)}^{3}-f\sin\tau =0,$$

(79) $$B_{1}^{\prime \prime }+\Omega_{2}^{2}B_{2}+\gamma_{1}B_{0}-e_{2}A_{0}-a_{2}B_{0}^{3}+b_{2}{\left(B_{0}-A_{0}^{}\right)}^{3}=0\begin{array}{cc} , & A_{1}(0)=B_{1}(0)=A_{1}^{\prime }(0)=B_{1}^{\prime }(0)=0 \end{array}$$

(80) $$\begin{array}{ll} p^{2}: & \;\;\;\;\;\;\;\;\;\;\;\;\;\;\;\;\;\;\;\;\;\;\;\;\;\;\;\;\;\;\;\;\;\;\;\;\;\;\;A_{2}^{\prime \prime }+\Omega_{1}^{2}A_{2}+\lambda_{2}A_{0}+\lambda_{1}A_{1}+\xi (1-\cos2\tau )A_{1}^{\prime \prime }+2\xi (\sin2\tau )A_{1}^{\prime }-\\ & \;\;\;\;\;\;\;\;\;\;\;\;\;\;\;\;\;\;\;\;\;\;\;\;\;\;\;\;\;\;\;\;\;\;\;\;-d_{2}(\cos2\tau )A_{1}-e_{1}B_{1}-Q_{1}(3A_{0}^{2}A_{1})-3b_{1}{\left(B_{0}-A_{0}\right)}^{2}(B_{1}-A_{1})=0, \end{array}$$

(81) $$B_{2}^{\prime \prime }+\Omega_{2}^{2}B_{2}+\gamma_{2}B_{1}+\gamma_{2}B_{0}-e_{2}A_{1}-3a_{2}B_{0}^{2}B_{1}+3b_{2}{\left(B_{0}-A_{0}^{}\right)}^{2}(B_{1}-A_{1})=0\begin{array}{cc} , & A_{2}(0)=B_{2}(0)=A_{2}^{\prime }(0)=B_{2}^{\prime }(0)=0 \end{array}$$

The solutions of Equations (76) and (77) are

(82) $$A_{0}(\tau )=A\cos\Omega_{1}\tau \begin{array}{cc} ; & B_{0}(\tau )=B\cos\Omega_{2}\tau \end{array}$$

Substituting Equation (82) into Equations (78) and (79), and avoiding secular terms we have

(83) $$\lambda_{1}=\xi \Omega_{1}^{2}+\frac{3}{4}a_{1}A^{2}-\frac{3}{4}b_{1}(A^{2}+2B^{2})$$

(84) $$\gamma_{1}=\frac{3}{4}a_{2}B^{2}-\frac{3}{4}b_{2}(2A^{2}+B^{2})$$

The solutions of Equations (78) and (79) are, respectively:

(85) $$\begin{array}{l} A_{1}(\tau )=\alpha \cos\Omega_{1}\tau +\frac{f}{\Omega_{1}^{2}-1}(\sin\tau -\frac{\sin\Omega_{1}\tau }{\Omega_{1}})-\frac{(d_{2}+2\Omega_{1}+\xi \Omega_{1}^{2})A}{8(\Omega_{1}+1)}\cos(\Omega_{1}+2)\tau +\frac{(d_{2}-2\Omega_{1}+\xi \Omega_{1}^{2})A}{8(\Omega_{1}-1)}\cos(\Omega_{1}-2)\tau +\\ +\frac{e_{1}B}{\Omega_{1}^{2}-\Omega_{2}^{2}}\cos\Omega_{2}\tau -\frac{a_{1}A^{3}}{32\Omega_{1}^{2}\cos3\Omega_{1}\tau }+\frac{b_{1}A^{3}}{32\Omega_{1}^{2}}\cos3\Omega_{1}\tau +\frac{b_{1}B^{3}}{4(\Omega_{1}^{2}-9\Omega_{2}^{2})}\cos3\Omega_{2}\tau +\frac{3b_{1}B^{3}}{4(\Omega_{1}^{2}-\Omega_{2}^{2})}\cos\Omega_{2}\tau +\\ +\frac{3AB^{2}b_{1}}{16}[\frac{\cos(2\Omega_{2}+\Omega_{1})\tau }{\Omega_{2}(\Omega_{1}+\Omega_{2})}-\frac{\cos(2\Omega_{2}-\Omega_{1})\tau }{\Omega_{2}(\Omega_{1}-\Omega_{2})}]+\frac{3A^{2}Bb_{1}}{2(\Omega_{1}^{2}-\Omega_{2}^{2})}\cos\Omega_{2}\tau +\\ +\frac{3A^{2}Bb_{1}}{2}\left[\frac{\cos(\Omega_{2}-2\Omega_{1})\tau }{\left(3\Omega_{1}-\Omega_{2})(\Omega_{2}-\Omega_{1}\right)}-\frac{\cos(\Omega_{2}+2\Omega_{1})\tau }{\left(3\Omega_{1}+\Omega_{2})(\Omega_{2}+\Omega_{1}\right)}\right] \end{array}$$

where

(86) $$\alpha =\frac{(2-\xi )\Omega_{1}^{2}-d_{2}}{4(\Omega_{1}^{2}-1)}A+\frac{3b_{1}AB^{2}-12b_{1}A^{2}B-8b_{1}B^{3}-8e_{1}B}{8(\Omega_{1}^{2}-\Omega_{2}^{2})}+\frac{(a_{1}-b_{1})A^{3}}{32\Omega_{1}^{2}}-\frac{b_{1}B^{3}}{4(\Omega_{1}^{2}-9\Omega_{2}^{2})}-\frac{3b_{1}A^{2}B(3\Omega_{1}^{2}+\Omega_{2}^{2})}{\left(\Omega_{2}^{2}-\Omega_{1}^{2})(9\Omega_{1}^{2}-\Omega_{2}^{2}\right)}$$

and

(87) $$\begin{array}{l} B_{1}(\tau )=\alpha_{0}\cos\Omega_{2}\tau +\frac{e_{2}A\cos\Omega_{1}\tau }{\Omega_{2}^{2}-\Omega_{1}^{2}}-\frac{a_{2}B^{2}}{32\Omega_{2}^{2}}\cos3\Omega_{2}\tau +\frac{b_{2}B^{3}}{32\Omega_{2}^{2}}\cos\Omega_{2}\tau -\frac{b_{2}A^{3}}{4}\left(\frac{\cos3\Omega_{1}\tau }{\Omega_{2}^{2}-9\Omega_{1}^{2}}+\frac{3\cos\Omega_{12}\tau }{\Omega_{2}^{2}-\Omega^{1}}\right)+\frac{b_{1}AB^{2}\cos\Omega_{2}\tau }{2(\Omega_{2}^{2}-\Omega_{1}^{2})}-\\ -\frac{3b_{2}AB^{2}\cos(2\Omega_{2}+\Omega_{1})\tau }{4(\Omega_{1}^{2}+4\Omega_{1}\Omega_{2}+3\Omega_{2}^{2})}-\frac{3b_{2}AB^{2}\cos(2\Omega_{2}+\Omega_{1})\tau }{4(\Omega_{1}^{2}-4\Omega_{1}\Omega_{2}+3\Omega_{2}^{2})}+\frac{3b_{2}A^{2}B\cos(2\Omega_{1}+\Omega_{2})\tau }{16\Omega_{2}(\Omega_{1}+\Omega_{2})}-\frac{3b_{2}A^{2}B\cos(\Omega_{2}-2\Omega_{1})\tau }{16\Omega_{1}(\Omega_{2}-\Omega_{1})} \end{array}$$

where

(88) $$\alpha_{0}=-\frac{(2e_{2}+b_{2}B)A}{2(\Omega_{2}^{2}-\Omega_{1}^{2})}+\frac{(a_{2}-b_{2})B^{3}}{32\Omega_{2}^{2}}+\frac{b_{2}A^{3}(2\Omega_{2}^{2}-5\Omega_{1}^{2})}{2(\Omega_{2}^{4}-10\Omega_{1}^{2}\Omega_{2}^{2}+9\Omega_{1}^{4})}+\frac{3b_{2}AB^{2}(\Omega_{1}^{2}+3\Omega_{2}^{2})}{2(\Omega_{1}^{4}-55\Omega_{1}^{2}\Omega_{2}^{2}+9\Omega_{2}^{4})}+\frac{3b_{2}A^{2}B(\Omega_{1}^{2}+\Omega_{2}^{2})}{16\Omega_{1}\Omega_{2}(\Omega_{2}^{2}-\Omega_{1}^{2})}$$

Now, substituting Equations (82), (83) and (87) into Equations (80) and (81) and avoiding secular terms in the two equations, we obtain

(89) $$\begin{array}{l} \lambda_{2}=\frac{9a_{1}+3b_{1}}{4}A\alpha +\frac{\xi (\Omega_{1}+2)(\Omega_{1}+3)}{8(\Omega_{1}+1))}(d_{2}+2\Omega_{1}+\xi \Omega_{1}^{2})-\frac{\xi (\Omega_{1}-2)}{8}(d_{2}-2\Omega_{1}+\xi \Omega_{1}^{2})+\frac{d_{1}(d_{2}+(\xi -1)\Omega_{1}^{2})}{2(\Omega_{1}^{2}-1)}+\\ +\frac{2e_{1}b_{1}A(2B^{2}-3A^{2})+27b_{1}b_{2}(A^{2}+B^{2})(2B^{2}-3A^{2})+2b_{1}b_{2}(A^{2}-B^{2})(2B^{2}-3A^{2})}{8(\Omega_{2}^{2}-\Omega_{1}^{2})}+\frac{9a_{1}A^{2}}{4}+\frac{3a_{1}b_{1}A^{4}}{64\Omega_{1}^{2}-1}-\\ -3b_{1}B\left(\alpha_{0}+\frac{e_{2}A}{3\Omega_{1}^{2}}\right)+\frac{9b_{1}b_{2}A^{2}B^{2}(\Omega_{1}^{2}+\Omega_{2}^{2})}{16\Omega_{1}\Omega_{2}(\Omega_{2}^{2}-\Omega_{1}^{2})}-\frac{3b_{1}b_{2}A^{2}(A^{2}-B^{2})}{128\Omega_{1}^{2}}+\frac{9b_{1}b_{2}A^{2}B^{2}(3\Omega_{2}-5\Omega_{1})}{4(\Omega_{1}^{2}-\Omega_{2}^{2})(3\Omega_{1}-\Omega_{2})} \end{array}$$

(90) $$\gamma_{2}=-3\alpha_{0}a_{2}B-\frac{3b_{2}}{4}(B^{2}+2A^{2})+\frac{4e_{1}e_{2}+3e_{2}b_{1}(B^{2}+2A^{2})}{\Omega_{1}^{2}-\Omega_{2}^{2}}$$

Substituting Equations (83), (89) and (90) into Equation (72) one gets a system of two equations with unknown Ω_1_ and Ω_2_. This system can be solved numerically.

**Figure 4:**
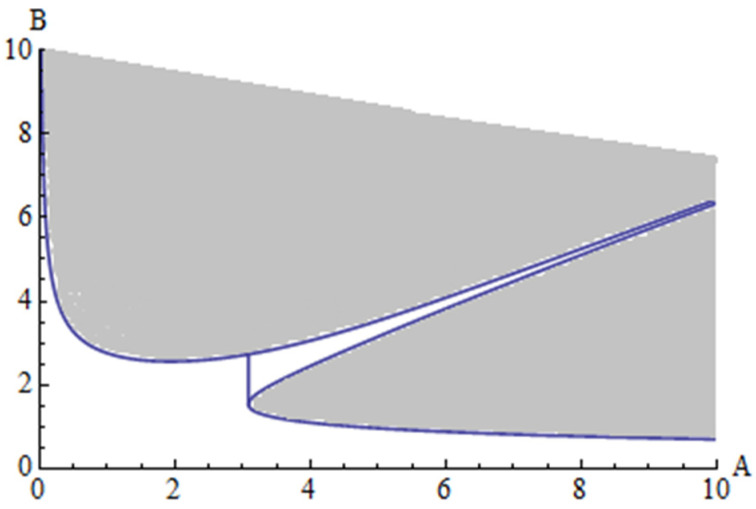
Stability domain for case 1 (shadow area): a_1_ = 2, a_2_ = 2.5, e_1_ = 3, e_2_ = 3.3.

**Figure 5:**
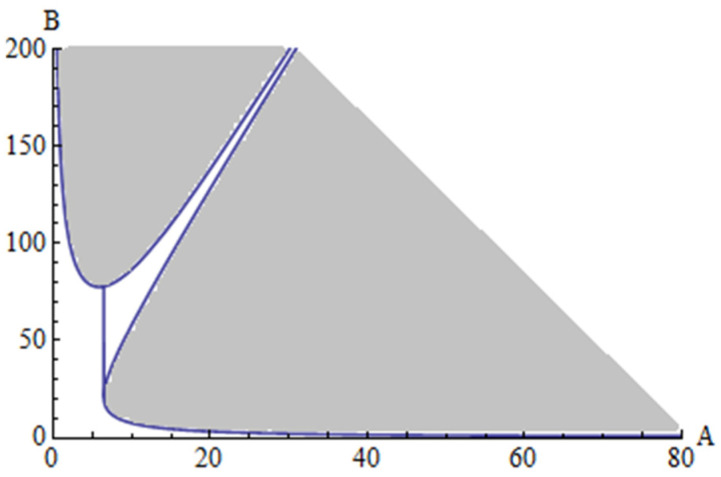
Stability domain for case 2 (shadow area): a_1_ = 0.2, a_2_ = 0.25, e_1_ = 0.3, e_2_ = 0.33.

The stability boundaries obtained up to the p^2^ order are presented in Figure 4 and Figure 5 for two particular cases, with different quantities. In Figure 4 is presented the stability domain for the case when a_1_ = 2, a_2_ = 2.5, e_1_ = 3, e_2_ = 3.3, while in Figure 5 is presented the stability domain for the case when a_1_ = 0.2, a_2_ = 0.25, e_1_ = 0.3, e_2_ = 0.33.

The boundary stability of the first tube is obtained from Equation (72). This boundary curve is $4-\lambda_{1}-\lambda_{2}=0$, where λ_1_ and λ_2_ are given by Equations (83) and (89). On the other hand, the boundary curve of the second tube can be obtained from $4-\gamma_{1}-\gamma_{2}=0$, where γ_1_ and γ_2_ are given by Equations (84) and (90). The area of stability for the first tube is given by $4-\lambda_{1}-\lambda_{2}>0$ and for the second tube the area of stability is given by $4-\gamma_{1}-\gamma_{2}>0$.

## 7. Conclusions and Discussion

After carrying out an analysis of nonlinear vibration of double-walled carbon nanotubes subjected to mechanical impact, which is an important research direction in the field of new materials and has a large domain of applicability in engineering practice, the following conclusions can be highlighted.

- We studied the nonlinear vibration of DWCNTs embedded on Winkler–Pasternak foundation, interacting through van der Waals forces, for which the governing differential equations contain nonlinear terms of order three and accordingly such equations are very difficult to solve by exact or approximate methods.
- For the first time is considered the simultaneous presence of nonlinearities induced by the Winkler–Pasternak foundation, the curvature of beams and the nonlinear van der Waals force, and also the effect of discontinuities marked by the presence of the Dirac function, which substantially complicates the governing equations.
- The OAFM was successfully applied to obtain an approximate analytical solution near the primary resonance. In contrast to any other known techniques, the proposed original approach is based on the presence of so-called auxiliary functions and optimal convergence-control parameters which assure high accuracy of the approximate analytical solutions. These parameters are determined by means of rigorous mathematical procedure. The initial and the first iteration are constructed by an original technique and the initial nonlinear governing equations are reduced to only two linear differential equations. Our procedure is easy to apply and very accurate using only the first iteration.
- A numerical example is developed in order to emphasize the accuracy of the proposed analytical solution. In the considered example, it is observed that the variables Q_1_ and Q_2_ describing the motion have completely different amplitudes at relatively close frequencies. The amplitude of Q_2_ is 20 times higher than the amplitude of Q_1_.
- A stability analysis of the considered model is developed by means of a homotopy perturbation method (HPM) using the condition of existence of the two frequencies of the system. This procedure enables splitting the parameter planes in stable and unstable regions and determines the borderline semianalytically.
- It is observed that an increasing of the values of the constitutive parameters a_1_, a_2_, e_1_, and e_2_ substantially reduces the area of stability for the considered system. Increasing these values tenfold led to a reduction of approximately 90% of the area of stability.

All the findings emphasized by this research are of important physical significance in guiding the design of advanced devices in the field of nanostructural applications in which nanotubes act as basic elements, with possible applications in nanosensors, nanoresonators, nanoswitches, nanoresistors, nanomotors, nanorobots, and so on.

## Data Availability

Not applicable.

## Abbreviations

CNTs	carbon nanotubes
SWCNTs	single-walled CNTs
DWCNTs	double-walled CNTs
SWBNNT	single-walled boron nitride nanotubes
OAFM	optimal auxiliary functions method
HPM	homotopy perturbation method
